# Analysis of the Intestinal Lumen Microbiota in an Animal Model of Colorectal Cancer

**DOI:** 10.1371/journal.pone.0090849

**Published:** 2014-03-06

**Authors:** Qingchao Zhu, Zhiming Jin, Wen Wu, Renyuan Gao, Bomin Guo, Zhiguang Gao, Yongzhi Yang, Huanlong Qin

**Affiliations:** Department of Surgery, The Sixth People’s Hospital Affiliated to Shanghai Jiao Tong University, Shanghai, China; University of Aberdeen, United Kingdom

## Abstract

Recent reports have suggested that multiple factors such as host genetics, environment and diet can promote the progression of healthy mucosa towards sporadic colorectal carcinoma. Accumulating evidence has additionally associated intestinal bacteria with disease initiation and progression. In order to examine and analyze the composition of gut microbiota in the absence of confounding influences, we have established an animal model of 1, 2-dimethylhydrazine (DMH)-induced colon cancer. Using this model, we have performed pyrosequencing of the V3 region of the 16S rRNA genes in this study to determine the diversity and breadth of the intestinal microbial species. Our findings indicate that the microbial composition of the intestinal lumen differs significantly between control and tumor groups. The abundance of Firmicutes was elevated whereas the abundance of Bacteroidetes and Spirochetes was reduced in the lumen of CRC rats. Fusobacteria was not detected in any of the healthy rats and there was no significant difference in observed Proteobacteria species when comparing the bacterial communities between our two groups. Interestingly, the abundance of Proteobacteria was higher in CRC rats. At the genus level, *Bacteroides* exhibited a relatively higher abundance in CRC rats compared to controls (14.92% vs. 9.22%, *p*<0.001). Meanwhile, *Prevotella* (55.22% vs. 26.19%), *Lactobacillus* (3.71% vs. 2.32%) and *Treponema* (3.04% vs. 2.43%), were found to be significantly more abundant in healthy rats than CRC rats (*p*<0.001, respectively). We also demonstrate a significant reduction of butyrate-producing bacteria such as *Roseburia* and *Eubacterium* in the gut microbiota of CRC rats. Furthermore, a significant increase in *Desulfovibrio, Erysipelotrichaceae* and *Fusobacterium* was also observed in the tumor group. A decrease in probiotic species such as *Ruminococcus* and *Lactobacillus* was likewise observed in the tumor group. Collectively, we can conclude that a significant difference in intestinal bacterial flora exists between healthy rats and CRC rats.

## Introduction

Each year, approximately 1.2 million individuals are diagnosed with colorectal cancer (CRC) worldwide [1]. CRC is the third most common cancer in men and the second most common in women with the majority of cases occurring in the developed world. A complex cellular community exists within a malignant tumor. This community was constituted by oncogenically transformed cells, non-neoplastic cells such as stromal and immune cells, and microbes such as bacteria and viruses in some cases [2]. Many types of cancer are associated with infectious agents and these cancers tend to occur in mucosal tissues that have high-level exposure to microbes. Some believe that up to one-fifth of all cancers are caused or promoted by infectious agents [3]. For example, cervical cancer and gastric cancers can be caused by human papillomaviruses and the bacterium, *Helicobacter pylori*, respectively [4].

It is evaluated that the total cell number of different bacteria species in human gastrointestinal tract is 10^14^, which is more than 10-fold the number of eukaryotic human cells [5] and perhaps as many as 10-fold more viruses. In a healthy gut, the normal bacterial flora maintains homeostasis with the host [6]. However, changes in bacterial populations and their metabolic products have been linked to several diseases including ulcerative colitis, Crohn’s disease and CRC [7]–[9]. And there are growing reports that the gut microbiota plays important role in the development of the colon carcinogenesis [10]. For instance, in animal model studies, mutant mice that are genetically susceptible to CRC were found to develop significantly fewer tumors when maintained in germ-free environments [11]. Wei and his colleagues determined the structure changes of gut microbiota of rats developing precancerous mucosal lesions induced by carcinogen DMH treatment, and demonstrated that the abundance of *Ruminococcus*-like and *Allobaculum*-like bacteria were increased in the feces of DMH-treated rats [12]. Moore and co-workers also reported that 15 bacterial species from human fecal flora were significantly associated with a high risk of colon cancer and 5 were associated with low risk of colon cancer [13]. In addition, Bacteroides and Bifidobacterium were most strongly associated with increased risk in their study of Caucasians, Japanese, Hawaiian, and African patients. These studies preliminarily demonstrated that there was a close relationship between the gut microbiota and the development of CRC [13]. However, no clear single bacterial species were identified as risk factors for CRC because about 80% of human bacteria were considered uncultivable [14]. To overcome this problem and investigate microbial diversity, researchers have turned to the field of metagenomics [15]. In 2011, there were four high-resolution maps referring to the association between human colonic dysbiosis and CRC emerged in succession, which reported by three independent groups [2], [8], [16], and several bacterial species were found to be preferentially inhabit either tumor tissues or surrounding non-tumor tissues. The enrichment of *Fusobacterium spp.* in the tumor samples was strikingly similar with those documented CRC microbiomes. In particular, one isolate of Fusobacteria (CC53), was demonstrated to have invasiveness in cultured colonic adenocarcinoma-2(Caco-2) cells [16]. These studies also reported a relative abundance of *Bacteroidaceae*, *Streptococcaceae*, *Fusobacteriaceae*, *Peptostreptococcaceae*, *Veillonellaceae*, and *Pasteurellaceae* in cancerous tissues compared to the normal intestinal lumen [17]. Furthermore, in order to establish colorectal cancer-related dysbiosis, Sobhani *et al*. investigated the stool microbiota of normal and colon cancer patients using pyrosequencing and subsequent principal component analysis (PCA) [18]. And they detected a composition change in the gut microbiota of CRC patients. In particular, *Bacteroides* and *Prevotella* species were found to be more abundant in cancer patients than in control subjects. Taken together, these studies demonstrated that the gut microbiota might play an important role in CRC development.

Host genetics, environment and diet have a dramatic effect on the host microbiota of individuals from different countries [19]–[20]. Therefore, we can observe that variation exists in the composition of gut microbiota leading to clinical associations between bacterial infection and CRC. In this study we have established an animal model of 1,2-dimethylhydrazine (DMH)-induced colon cancer and performed pyrosequencing of 16S rRNA genes to compare the microbiota within the intestinal lumen of CRC rats and healthy controls. We have additionally identified bacterial phylotypes that may serve as potential biomarkers in CRC development.

## Materials and Methods

### Animals and Reagents

Four-week-old male Wistar rats (180–200 g) purchased from Shanghai Shriek Laboratory Animal Corporation (Shanghai, China) were used for this study. All animals were housed in plastic cages (with four or five rats/cage) under controlled conditions of humidity (44±5%), light (12 h light/dark cycle) and temperature (22±2°C). 1, 2-Dimethylhydrazine (DMH) was purchased from Sigma-Aldrich (St. Louis, MO, USA). DMH was prepared fresh before use in 1 mM EDTA-saline and pH adjusted to 7.0 using dilute NaOH solution.

### Experimental Procedures

Forty 4-week-old male Wistar rats were divided into two groups: a DMH induced-tumor group (TG, n = 30) and a non-DMH-induced-tumor group (control group, CG, n = 10). Animals were acclimatized to rodent diet and water *ad libitum* for 1 week. After the acclimation, the rats from TG were intraperitoneally (i.p.) injected with DMH (40 mg/kg) once a week for 10 consecutive weeks. The remaining 10 rats were intraperitoneally injected with EDTA – normal saline as controls. The animal weights were recorded once a week throughout the experimental period. Beginning at the 12th week of the protocol, three rats were euthanized every 2 weeks in order to examine the formation of colon tumors. The animals were anaesthetized with Ketamine 100 mg/kg and Xylazin 15 mg/kg body weight i.p. under aseptic conditions. The entire colon was surgically removed and opened longitudinally. Stool samples were collected and frozen immediately in liquid nitrogen. The stool samples were later transferred to −80°C until DNA extraction was performed. The colon was photographed and the total number of tumors was counted. For histological examination, colon tumors were separately excised and fixed in 10% neutral phosphate-buffered formalin.

### Histological Examination

For histological examination, the fixed tissues were embedded and sectioned at 5 µm intervals. Tissue was stained with standard hematoxylin and eosin for light microscopic examination. Tissue sections were reviewed by two independent pathologists in a blind fashion. Any discrepancy between these two investigators was resolved through the reevaluation by the third pathologist until consensus of opinion was reached.

### Bacterial DNA Extraction

Nucleic acids were extracted from each stool samples using a method modified from the manufacturer’s guidelines for the QIAamp DNA Stool Mini Kit (Qiagen, Hilden, Germany). The quantity of DNA was determined by Synergy 2 Multi-Mode Microplate Reader (BioTek, US). Integrity and size of DNA was determined by 1% (w/v) agarose gel electrophoresis. All DNA samples were stored at −20°C until use. Tubes containing only the QIAamp DNA Stool Mini Kit extraction controls were included throughout the lysis and PCR steps to serve as negative controls.

### PCR and 454 Pyrosequencing

The following universal 16S ribosomal RNA primers: (27F: 5′-AGAGTTTGATCCTGGCTCAG-3′, 533R: 5′-TTACCGCGGCTGCTGGCAC-3′) correspond to the V3 positions of the 16S rRNA gene, with a sample barcode sequence and the FLX Tianium adapters were used to amplify the V3 region of each fecal sample by polymerase chain reaction. PCR was performed with 10 ng template, 0.4 µl FastPfu Polymerase (TransGen Biotech, China), 4 µl 5×FastPfu buffer, 2 µl dNTPs (2.5 mM each, Takara Bio, Japan), 0.4 µl forward primers (5 µM) and 0.4 µl reverse primers (5 µM) on an ABI GeneAmp® 9700 cycler. The cycling parameters were as follows: 5 min of denaturation at 95°C followed by 25 cycles of 30 seconds at 95°C (denaturation), 30 seconds for annealing at 55°C and 30 seconds at 72°C (elongation), with a final extension at 72°C for 5 min. Triplicate PCR reactions were performed on each sample. Amplified products from stool samples were verified by gel electrophoresis using 5 µl of the PCR reaction mixture in a 2.0% agarose gel. The PCR products were purified by using the AxyPrepDNA Gel extraction kit (Axygen, US) and quantified on QuantiFluor™-ST Fluorometer (Promega, US). The products from different samples were mixed at equal ratios for pyrosequencing using the Roche GS FLX 454 Sequencer according to the manufacturer’s instructions.

All pyrosequencing reads were then removed of their primers, barcodes, and adaptor sequences, and further screened and filtered according to the standards for quality control as follows: the elimination of sequences that did not perfectly match the proximal PCR primer (over two mismatches to the primers), those with short sequencing length (less than 200 nt) sequences that contained mononucleotide repeats of 6 nt, sequences with ambiguous characters, or sequences with a read quality score <25. Finally, a total of 197,911 high-quality sequences from twenty samples were produced, which accounted for 62.9% of valid sequences according to barcode- and primer-sequence filtering.

### Bioinformatic Analysis of Sequencing Data

The sequences were aligned using SILVA (http://www.arb-silva.de/) database, and delineation of operational taxonomic units (OTUs) was conducted with Mothur at 97% cutoff according to their pairwise distances. Then we conducted the analysis of the Good’s coverage, diversity estimators (Shannon and Simpson), richness estimators (Chao1 and Ace), and rarefaction curve using the Mothur software package (http://www.mothur.org/wiki/Main_Page) at the 80% confidence level [21]. The heatmap was constructed on genus information with the heatmap 2 function in R vegan package. In addition, Bray-Curtis similarities were used to construct a cluster dendrogram. We also conducted the Unweighted Unifrac distance metrics analysis using OTUs from each sample, and performed the principal component analysis in terms of the matrix of distance. A metagenomic biomarker discovery approach was employed with LEfSe [linear discriminant analysis (LDA) coupled with effect size measurement] which performed a nonparametric Wilcoxon sum-rank test followed by LDA analysis using online software (http://huttenhower.sph.harvard.edu/galaxy/) to assess the effect size of each differentially abundant taxon [22].

### Statistical Analysis

The *t*-test and Mann-Whitney test were performed using SPSS version 19.0 for Windows.

### Ethics Statement

The experimental protocol was reviewed and approved by the Animal Care and Use Committee and the Ethics Committee of the Sixth People’s Hospital Affiliated to Shanghai Jiao Tong University.

### Data Access

The 16S sequence information generated in this study has been submitted to the NCBI Sequence Read Archive under accession number SRA098098.

## Results

### Animal Models and Tumor Formation

**Figure 1:**
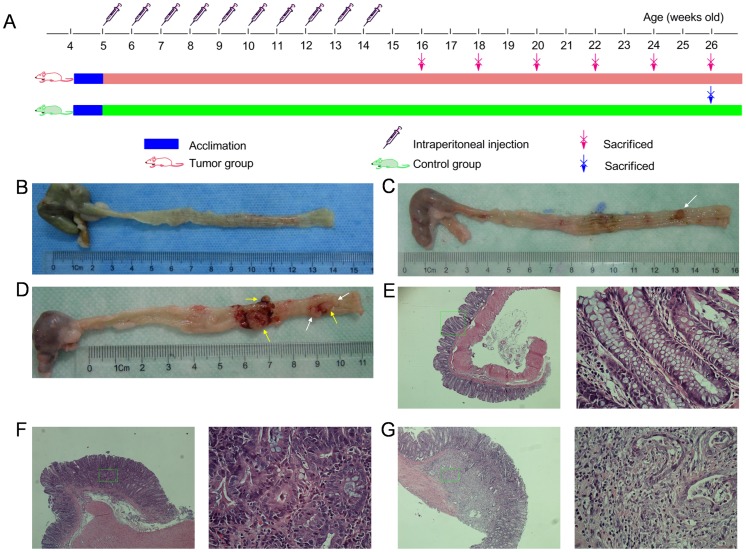
Experimental protocol, representative pictures of colon tumors and photomicrographs showing the pathological characteristic. (A) Experimental procedures. (B) Normal colon. (C) Adenoma (white arrow). (D) Adencarcinoma (yellow arrow). The bloodstain around the tumor was caused by tumor ulcer instead of our dissection. Representative photomicrographs showing the normal mucosa (E), adenoma (F) and adenocarcinoma (G) were magnified in 40×(left), and each of the right micrograph (400x) highlighted the district from the corresponding green rectangle.

According to the experimental protocol (Figure 1A), we euthanized three DMH-treated rats every two weeks beginning in the 12th week. Adenoma was first found in the 12th week in one of three animals euthanized. The majority of adenomas, however, were found between the 14th and 18th weeks (7/9). Unfortunately, two DMH-treated rats died in the 19th week of colon obstruction and cachexia. In the samples from the 20th week, we found that two rats demonstrated adenocarcinoma (2/3) with occasionally adenoma within the tissue. As a result, we decided to euthanize the remainder of the DMH-treated rats as well as those belong to the control group in the 22nd week in order to keep the rats’ life cycle in concordance. At necropsy, we found pathologically confirmed colon adenocarcinoma successfully induced in eleven DMH-treated rats (11/13) with no evidence of organ metastasis. The remaining two rats displayed no signs of tumor formation. However, one of the animals was found to have an incomplete colon obstruction, therefore only ten DMH-treated rats were included in our final study group. Meanwhile, all rats in the control group survived to the 22th week. The average body weight in the ten selected DMH-treated rats was 343.5±10.74 g and 359.3±7.61 g in the control animals with no statistical significance (*p* = 0.59). Details of DMH-induced tumors are summarized in Table 1 and shown in Figure 1B–G.

**Table 1:** Colon tumor incidence, pathological characteristics, numbers in both DMH-treated and control groups.

Week	*n*	Incidence *n* (%)		Pathological characteristics (*n*)		Tumor numbers (*n*)		
		Control	DMH	Ad	Ca	1	2	>2
12th	3	0 (0)	1 (33.3)	1	0	1	0	0
14th	3	0 (0)	2 (66.7)	2	0	1	0	1
16th	3	0 (0)	2 (66.7)	2	0	0	1	1
18th	3	0 (0)	3 (100.0)	3	0	1	0	2
20th	3	0 (0)	3 (100.0)	3*	2	0	0	3
22th	13	0 (0)	11 (84.6)	9*	11	1	2	8
Total	28	0 (0)	22 (78.6)	18	13	4	3	15

Pathological characteristics, tumor numbers referred to data from DMH-treated group because no tumor data was available in control group. (*n*): number of rats; Ad: adenoma; Ca: carcinoma; *the number of rats will be summarized here no matter there are colonic carcinomas exist in DMH-treated animals.

### Characteristics of 454 Pyrosequencing

In total, 314,880 valid sequences were obtained from all 20 samples, with an average of 15,744 sequences per sample. The resulting sequences were processed using Seqcln (http://sourceforge.net/projects/seqclean/) and Mothor [23]. After removing low quality sequences (<Q25) and sequences shorter than 200 bp, with homopolymers longer than six nucleotides, and containing ambiguous base calls or incorrect primer sequences, a total of 197,911 high-quality sequences were produced with an average length of 481 bp per sequence. Sequences were aligned against the silva database (SSU111 version: http://www.arb-silva.de/) using k-mer searching (http://www.mothur.org/wiki/Align.seqs). Potentially chimeric sequences were detected using UCHIME (http://drive5.com/uchime) and removed. The remaining reads were pre-clustered (http://www.mothur.org/wiki/Pre.cluster) and then clustered using uncorrected pairwise algorithm. The detailed characteristics of each sample are found in Table 2. In addition, Operational taxonomic units were defined as sharing >97% sequence identity using Furthest neighbor method (http://www.mothur.org/wiki/Cluster). The total number of OTUs at 97% similarity level was 41,923, with an average of 2096 OTUs per sample.

**Table 2:** Statistics of valid sequences, trimed sequences and corresponding percentage in each sample.

Sample ID	Valid	Trimed	Percent (%)	Sample ID	Valid	Trimed	Percent (%)
CGS_1	12 474	7710	61.81	TGS_1	15644	9594	61.33
CGS_2	14 759	9129	61.85	TGS_2	15913	10645	66.89
CGS_3	12261	7595	61.94	TGS_3	15280	9702	63.49
CGS_4	17084	10416	60.97	TGS_4	13125	8402	64.02
CGS_5	17108	10274	60.05	TGS_5	13938	8759	62.84
CGS_6	15783	9850	62.41	TGS_6	15723	9244	58.79
CGS_7	16300	8566	58.69	TGS_7	15815	10273	64.96
CGS_8	13886	8662	62.38	TGS_8	18647	12271	65.81
CGS_9	14128	8689	61.50	TGS_9	15833	10606	66.99
CGS_10	26200	16853	64.32	TGS_10	14979	9671	64.56
Mean	15998.3	9774.4	61.59	Mean	15489.7	9916.7	63.97

Sample ID: CGS, control group stool; TGS, tumor group stool.

The mean value of Good’s coverage for each group was over 80%, indicating that the 16S rRNA sequences identified in the two groups represent the majority of bacteria present in the study samples. Whereas we didn’t observe the plateau of the refraction curve (Figure S1A) with the current sequencing, the Shannon diversity estimates of all samples had already reached stable values at this sequencing depth, which suggests that, although identification of new phylotypes would be expected from additional sequencing, the range of diversity within the samples had been captured (Figure S1B, C, D). Statistically significant differences were seen in the Shannon indices between the tumor group and control group (5.92±0.30 vs. 6.17±0.20, *p* = 0.042, Figure S1E). Differences demonstrate that higher diversity could be found in the noncancerous intestinal lumen of rats from the control group which is confirmed by Simpson’s diversity index (0.024±0.013 vs. 0.013±0.007, *p* = 0.037, Figure S1F). The estimators of community richness (Chao1 and Ace) and detailed characteristics of each sample are shown in Table S1.

### Comparison of Gut Microbiota between Control Group and Tumor Group

**Figure 2:**
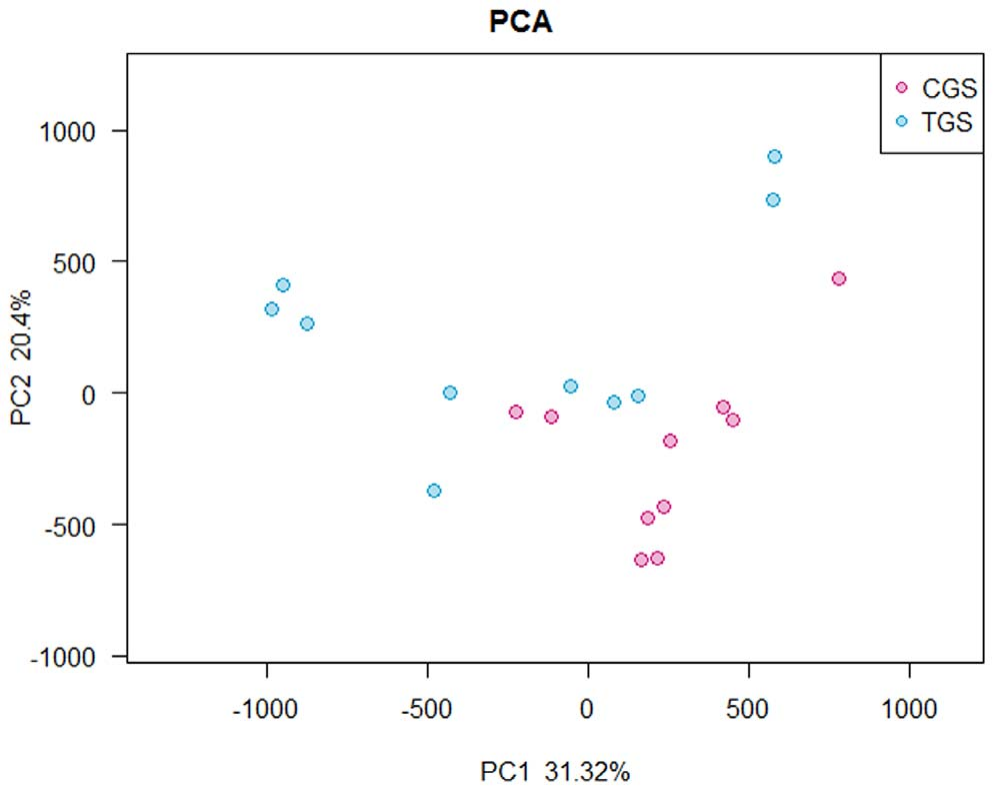
Principal component analysis (PCA) scores plot based on the relative abundance of OTUs (97% similarity level). Each symbol represents a sample. Red circles represent healthy rats; Blue circles represent CRC rats.

**Figure 3:**
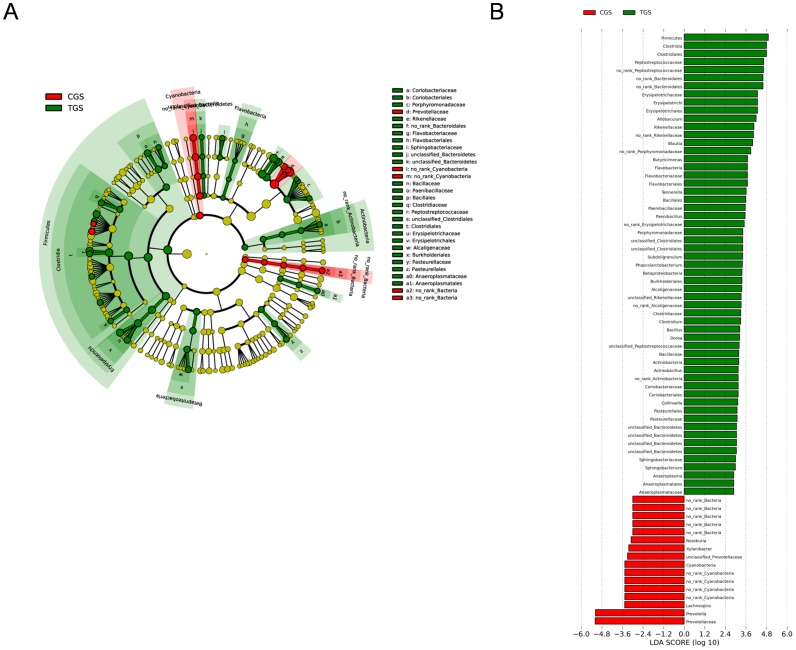
Different structures of gut microbiota between healthy rats and CRC rats. (A) Taxonomic representation of statistically and biologically consistent differences between healthy rats and CRC rats. Differences are represented by the color of the most abundant class (red indicating control group, green tumor group and yellow non-significant). The diameter of each circle’s diameter is proportional to the taxon’s abundance. (B) Histogram of the LDA scores for differentially abundant genera. Cladogram was calculated by LefSe, and displayed according to effect size.

The microflora and compositions of two groups were analyzed and compared through the relative abundance of OTUs by using the unweighted Unifrac distance matrix for each group. Subsequent results of PCA exhibited that there was significant difference in bacterial community composition between healthy rats and CRC rats using the first two principal component scores of PC1 and PC2(31.32% and 20.4% of explained variance, respectively) (Figure 2). In addition, LEfSe was performed to obtain the cladogram representation and the predominant bacteria of the microbiota within the two groups, which is shown in Figure 3A. We also showed the greatest differences in taxa between the two communities in Figure 3B. *Peptostreptococcaceae*, *Erysipelotrichales*, *Coriobacteriaceae* and *Porphyromonadaceae* were enriched in CRC rats, whereas *Roseburia* and *Prevotella* were enriched in healthy rats, all of which were key phylotypes involved in the segregation of intestinal microbiota in CRC and healthy rats in accordance with the LEfSe analysis.

### Comparisons of Gut Microbiota at Different Levels between Healthy Rats and CRC Rats

**Figure 4:**
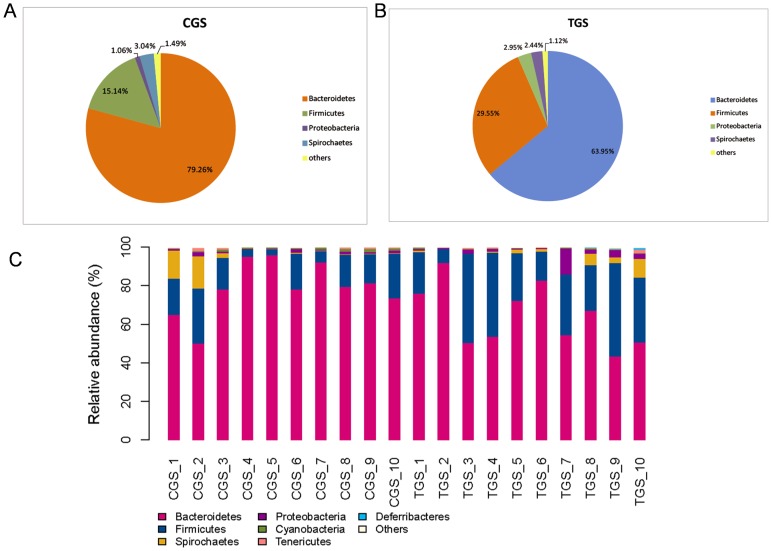
Relative contributions of dominant phyla in the intestinal lumen microbiota. (A) healthy rats, (B) CRC rats. Histogram represents the relative abundance of bacterial phyla in microbiota of each sample (C). “Others” represents the unclassified bacteria, Actinobacteria, Cyanobacteria, Deferribacteres, Elusimicrobia, Fusobacteria and Tenericutes.

We studied the bacterial communities in stool from the intestinal lumen of rats with or without CRC. The different phyla and genera were assessed by taxonomic assignment of all sequences and the overall microbial composition for each group at the phylum level is shown in Figure 4A, B. According to the taxonomic results, we demonstrated that Bacteroidetes, accounting for 79.26% and 63.95% of the gut microbiota in healthy and CRC rats respectively, was the most predominant phylum in our study. And Firmicutes were the secondary phylum with the proportion of 15.14% and 29.55%, respectively. Finally, Spirochaetes and Proteobacteria constituted the third most abundant phyla, contributing 3.04% and 1.06% in healthy rats, and 2.44% and 2.95% in CRC rats, respectively. The composition of dominating phyla is shown in Table S2. We find that the microbial composition shows high inter-individual variability (Figure 4C). Firmicutes accounted for 3.39%–48.35%, and Bacteroidetes 43.24%–95.79% among all individual animals (Table 3). Except that, the abundance of Bacteroidetes and Cyanobacteria were higher in the gut microbiota of healthy rats than that in CRC rats, and the difference showed statistically significance (*p* = 0.044 and 0.003, respectively) (Figure S2A, B). Whereas, Firmicutes and Actinobacteria were significantly less abundant microbiota in healthy rats (*p* = 0.01 and 0.035, respectively) (Figure S2C, D). From our data no Fusobacteria was detected in any of the healthy rats (Figure S2E) and there was no significant difference in Proteobacteria (*p* = 0.175) (Figure S2F) when comparing the bacterial communities of the two groups, although its abundance was higher in CRC rats. Statistically significant differences between the tumor group and control group at the family level were also performed in our study. Difference in the relative abundance of Bacteroidetes (11.6% vs. 4.6%, *p* = 0.0029), Rikenellaceae (3.71% vs. 1.47%, *p* = 0.0008), and Peptostreptococcaceae (9.18% vs. 1.61%, *p* = 0.046) were significant between CRC rats and healthy rats, while there was an extremely lower level of Prevotellaceae (31.91% vs. 62.88%, *p* = 0.0027) and Cyanobacteria (0.3% vs. 0.82%, *p* = 0.003) in CRC rats compared with healthy rats.

**Table 3:** Relative contribution, median and range of the predominant phyla in the gut microbiota of healthy rats and CRC rats.

Phylum	Relative contribution (%)		Median, range (%)	
	CGS	TGS	CGS	TGS
Bacteroidetes	79.26	63.95	78.82, 49.95–95.79	60.64, 43.24–91.75
Firmicutes	15.14	29.55	16.48, 3.39–28.62	28.21, 7.26–48.35
Proteobacteria	1.06	2.95	0.91, 0.26–2.04	1.67, 0.42–13.31
Spirochaetes	3.04	2.44	0.39, 0.11–16.82	1.12, 0.03–9.74
Tenericutes	0.47	0.43	0.41, 0.05–1.60	0.33, 0.01–1.71
Cyanobacteria	0.82	0.30	0.87, 0.27–1.51	0.26, 0.05–0.75
Deferribacteres	0.004	0.13	0, 0–0.04	0, 0–1.01
Actinobacteria	0.02	0.12	0.02, 0–0.04	0.07, 0.02–0.48
Fusobacteria	0	0.01	0, 0	0, 0–0.07

Abbreviations: CGS, control group stool; TGS, tumor group stool. Relative contribution of a phylum in healthy rats or CRC rats was calculated as percentage of the sequences of this phylum to all sequences in this group.

**Figure 5:**
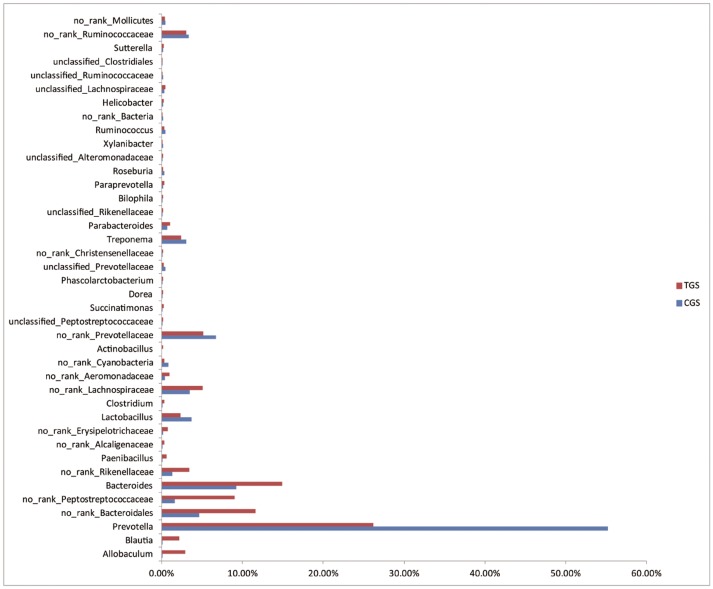
Relative abundance of significantly different genera between healthy rats and CRC rats. The Mann-Whitney test was used to evaluate the importance of comparisons between indicated groups.

At the genus level, we studied the microbial composition of each sample (Figure S3) and found them to be significantly different between the two groups. The ten most abundant genuses in the control group were *Prevotella*, *Bacteroides*, *Lactobacillus*, *Treponema*, *Parabacteroides*, *Anaerovibrio*, *Ruminococcus*, *Roseburia*, *Oscillospira* and *Sutterella*. However, *Prevotella*, *Bacteroides*, *Allobaculum*, *Treponema*, *Lactobacillus*, *Blautia*, *Parabacteroides*, *Paenibacillus*, *Anaerovibrio* and *Paraprevotella* were the ten most abundant genuses in tumor group, correspondingly (Figure S4). Interestingly, although *Prevotella* was the most abundant genus in both groups, the abundance of it was much higher in healthy rats. Statistically, *Bacteroides*, with over 1% of the total bacteria in stool, were relatively abundant in CRC rats. Of particular note, genera *Bacteroides. fragilis* was mainly found in CRC rats. Meanwhile, *Prevotella*, *Lactobacillus* and *Treponema* were found to be significantly higher in healthy rats than CRC rats (Figure 5). Genera *Desulfovibrio*, *Clostridium*, *Actinobacillus*, *Succinatimonas*, *Dorea*, *Phascolarctobacterium*, *Parabacteroides*, *Bilophila*, *Paraprevotella*, *Helicobacter* and *Paenibacillus* exhibited low abundance; however, they were all statistically enriched in stool of CRC rats compared to healthy rats. Moreover, genera *Roseburia, Eubacterium* and *Ruminococcus* were enriched in control group, and *Fusobacterium* was absent from healthy rats. The heatmap of the bacterial genus level also demonstrated the same phenomenon (Figure S5). Additional differences between the two groups can be found in Table S3.

## Discussion

The microbial population in the intestine is heterogeneous and complex. Laboratory rodents have been instrumental in helping researchers to unravel the complex interactions that mammals, including humans, have with their microbial commensals [24]. We have utilized a common animal model of CRC in this study to investigate the nature of microbial community structure in CRC compared to healthy animals. The studies performed in animal models could possibly have important implications relevant to human disease [25].

In this study, we compared the bacterial composition of the intestinal lumen of rats between healthy group and CRC group using the platform of Roche 454 sequencer. We observed significant differentiation of gut microbiota between healthy rats and carcinogen-treated rats that developed colonic carcinomas. We showed a relative higher abundance of Firmicutes, Proteobacteria and Actinobacteria in the intestinal lumen of CRC rats, and further showed that Bacteroidetes was less abundant in this group. This result is in accordance with similar findings by Zhao *et al.* [26] from human studies. It has been reported that the inflammatory bowel diseases such as Crohn’s disease and ulcerative colitis are known risk factors for colorectal cancer, and a significant reduction of the phylum Bacteroidetes was detected in these two diseases by researchers [27]–[28]. In further study, alterations among the genera of this phylum were also detected in our study. Especially, the finding that *B. fragilis* was increased in the intestinal lumen of CRC rats.

Previous studies have suggested that different *Bacteroides* strains may influence the health of the host through their colitogenic or probiotic potential. Waidmann and his colleagues had described the isolated B. vulgates strain with possible probiotic propertites that was able to ameliorate E. coli induced colitis development by an yet unknown mechanism in interleukin-2-deficient mice [29]. However, a human colonic commensal, enterotoxigenic *B. fragilis* have been demonstrated that the colonization of it in multiple intestinal neoplasia (Min) mice could result in a marked increase in colonic thickness, inflammation and visible colonic tumors which accompanied by the activation of STAT3 and the infiltration of T_H_17 inflammatory cells [30]. These research results are in agreement with our finding that *B. fragilis* was aplenty in CRC rats. In addition, Proteobacteria have also been reported to be increased in the microbiota of animals with experimentally induced colitis and patients suffering from IBD [31]. It might be relevant with the direct interaction between Proteobacteria and intestinal cells through bacterial secretion systems such as type III secretion system (T3SS) [32]. Furthermore, it has been reported that Firmicutes held the ability in enhancing energy harvest from diet [33]. The Firmicutes phylum contains relevant genera, including *Ruminococcus*, *Clostridium* and the butyrate producers *Eubacterium*, *Faecalibacterium* and *Roseburia* [34]. Butyrate is an important energy source for colonic epithelial cells, often preferred over circulatory glucose or glutamine. Up to 90% of butyrate is metabolized by colonocytes [35]. Here we show reduced abundance of *Roseburia* and *Eubacterium* in the gut microbiota of CRC rats. In two dietary intervention studies, population densities of *Roseburia* and *Eubacterium* were proven to have a strong correlation with fecal butyrate concentrations in response to altered carbohydrate supply [36], suggesting the importance of *Roseburia* and *Eubacterium* in the production of butyrate in vivo. Sengupta *et al.* [37] indicated that there may be some evidence that delivery of an adequate amount of butyrate to the intestinal mucosa may protect against early tumorigenic events. Therefore, the structure imbalance in this paper plays an important role in reducing the butyrate-producing bacteria in the gut of CRC rats.

Besides, the structure imbalance within the gut microbiota of CRC rats is significantly related with the increase in multiple potential pathogens and the decrease in probiotic species. It has been demonstrated that *Bacteroides* populations and more specifically those of *B. fragilis* produce a metalloprotease known as fragilysin in colon cancer patients, but not in controls. This report suggests that this subpopulation might favor carcinogenesis [38]. In addition, the serried *Desulfovibrio* reduces sulfate to produce hydrogen sulfide (H_2_S), which has been demonstrated to be capable of generating DNA damage that may be, in part, responsible for the generation of the genomic instability and the cumulative mutations observed in colorectal cancer [39]–[40]. In recent years, researchers have focused on *Fusobacterium*, Gram-negative bacteria, that usually multiplies in the oral cavity [41]. Certain groups have successively identified *Fusobacterium* out of tumors in patients with colorectal carcinoma [42]–[44] and reported that tumor tissue was characteristic of more abundant *Fusobacterium* than normal colon. More specifically, *Fusobacterium* has been reckoned as a pro-inflammatory organism [45] and found in IBD patients under higher abundance [42]. These results suggest *Fusobacterium* as a potential biomarker for colorectal carcinogenesis. From our results, the abundance of *Bacteroides*, *Desulfovibrio*, and *Fusobacterium* was found to be higher in the intestinal lumen of CRC rats, thus, we can hypothesize that these bacteria might play important roles in the development and progression of CRCs.

In summary, our study provides a significant probe into the CRC-associated microbiome in a rat CRC model mimicking human colorectal carcinogenesis. By comparing the gut microbial composition, we have identified a structure imbalance of microbiota in CRC rats, with butyrate producers and probiotics reduced and several potential pathogens increased, which may be a distinctive feature of human CRC. Thus we hypothesize that intestinal lumen microflora potentially affects CRC via co-metabolism or metabolic exchange with the host. Our results are also in accordance with findings that were conducted in recent human studies. Clearly many questions remain including whether the microbiological differences found in the present study are cause or consequence of tumor formation. We also need to provide more detailed information concerning mucosa-associated microbiota. Therefore, it can be predicted that further studies concerning these issues will enhance our understanding on driving forces of CRC and promote the development of novel microbiome-related diagnostic tools and therapeutic interventions.

## Supporting Information

Figure S1 **Rarefaction curves, Shannon diversity index curves and comparison of diversity indexes between two groups.** (A) Rarefaction cures of all samples. (B) Shannon diversity index curves of all samples. (C) Control group samples. (D) Tumor group samples. (E) Comparison of Shannon index. (F) Comparison of Simpson index.
(TIF)

Figure S2 **Statistically comparison of dominant phyla between control group and tumor group.** (A) Bacteroidetes. (B) Cyanobacteria. (C) Firmicutes. (D) Actinobacteria. (E) Fusobacteria. (F) Proteobacteria. CG, control group; TG, tumor group. (*p<0.05, **p<0.01, ns: not significant)
(TIF)

Figure S3 **Genus-level relative abundance of the microbiota from the intestinal lumen of healthy rats and CRC rats.** Genus-level classification demonstrates that most samples are dominated by the *Prevotella* and *Bacteroides*. “Others” represents a collective of the genus that whose relative abundance is very low in each sample.
(TIF)

Figure S4 **Genus abundance variation plot for the 10 most abundant genera of each group as determined by read abundance.** Blue represent control group; Red represent tumor group.
(TIF)

Figure S5 **Heatmap analysis of 100 most abundant genera in control group and tumor group.** The y axis is a cluster dendrogram, each row is a different phylotype. Clustering is indicative of abundance, not phylogenetic similarity. The abundance plot shows the proportion of 16S rRNA gene pyrosequences in each sample.
(TIF)

Table S1 **Mothur diversity indices of bacterial communities in samples from two groups.**
(DOCX)

Table S2 **Composition of dominating phyla.**
(DOCX)

Table S3 **Phylotypes significantly different between gut microbiota of healthy rats and CRC rats.**
(DOCX)
