# Supplementary figures and images for: Analysis of the Intestinal Lumen Microbiota in an Animal Model of Colorectal Cancer
Source: PLoS One. 2014 Mar 6;9(3):e90849. doi: 10.1371/journal.pone.0090849 (PMC3946251; doi:10.1371/journal.pone.0090849)

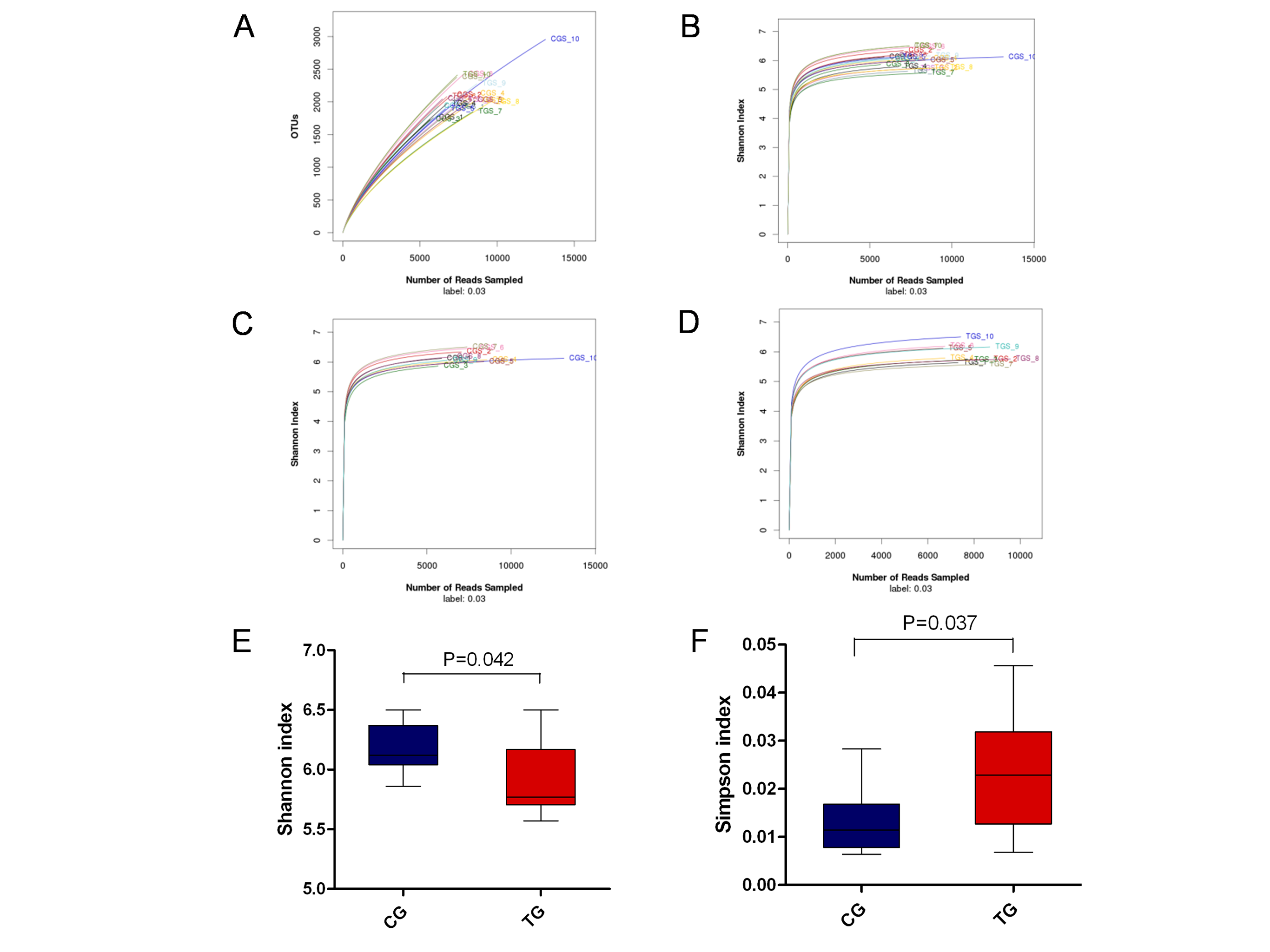

Supplement: Figure S1 — Rarefaction curves, Shannon diversity index curves and comparison of diversity indexes between two groups. (A) Rarefaction cures of all samples. (B) Shannon diversity index curves of all samples. (C) Control group samples. (D) Tumor group samples. (E) Comparison of Shannon index. (F) Comparison of Simpson index. (TIF) [file pone.0090849.s001.tif]

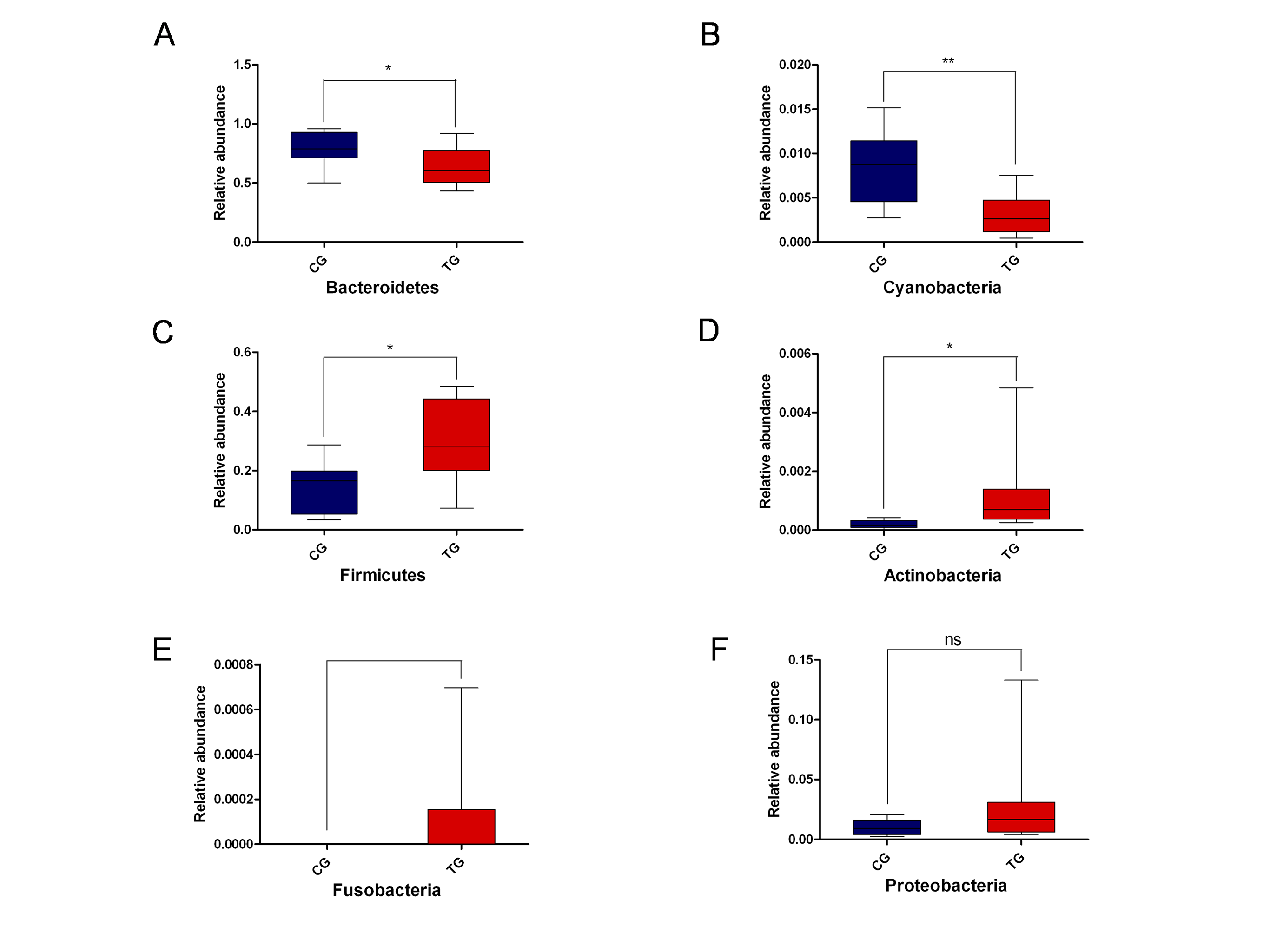

Supplement: Figure S2 — Statistically comparison of dominant phyla between control group and tumor group. (A) Bacteroidetes. (B) Cyanobacteria. (C) Firmicutes. (D) Actinobacteria. (E) Fusobacteria. (F) Proteobacteria. CG, control group; TG, tumor group. (*p<0.05, **p<0.01, ns: not significant) (TIF) [file pone.0090849.s002.tif]

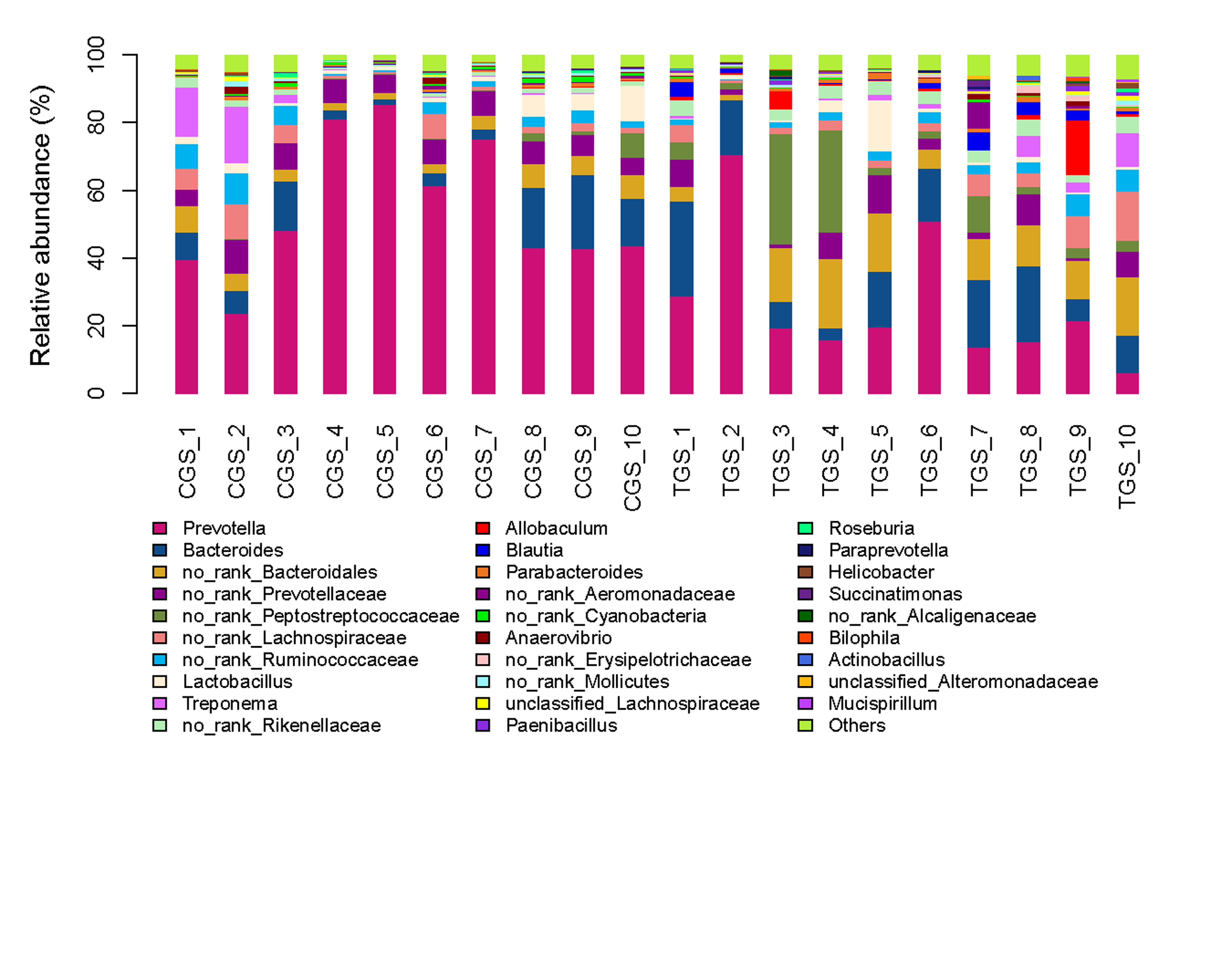

Supplement: Figure S3 — Genus-level relative abundance of the microbiota from the intestinal lumen of healthy rats and CRC rats. Genus-level classification demonstrates that most samples are dominated by the Prevotella and Bacteroides. “Others” represents a collective of the genus that whose relative abundance is very low in each sample. (TIF) [file pone.0090849.s003.tif]

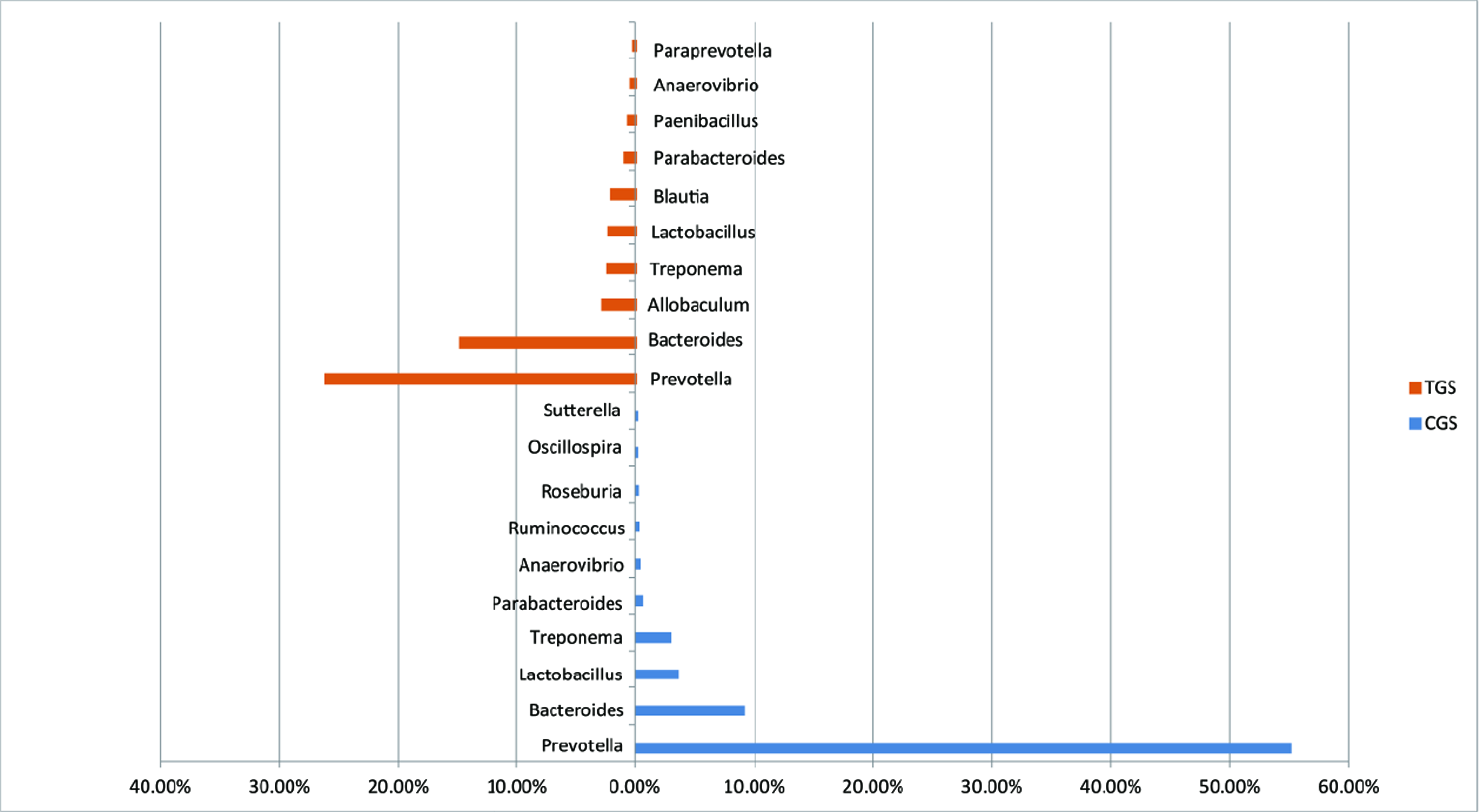

Supplement: Figure S4 — Genus abundance variation plot for the 10 most abundant genera of each group as determined by read abundance. Blue represent control group; Red represent tumor group. (TIF) [file pone.0090849.s004.tif]

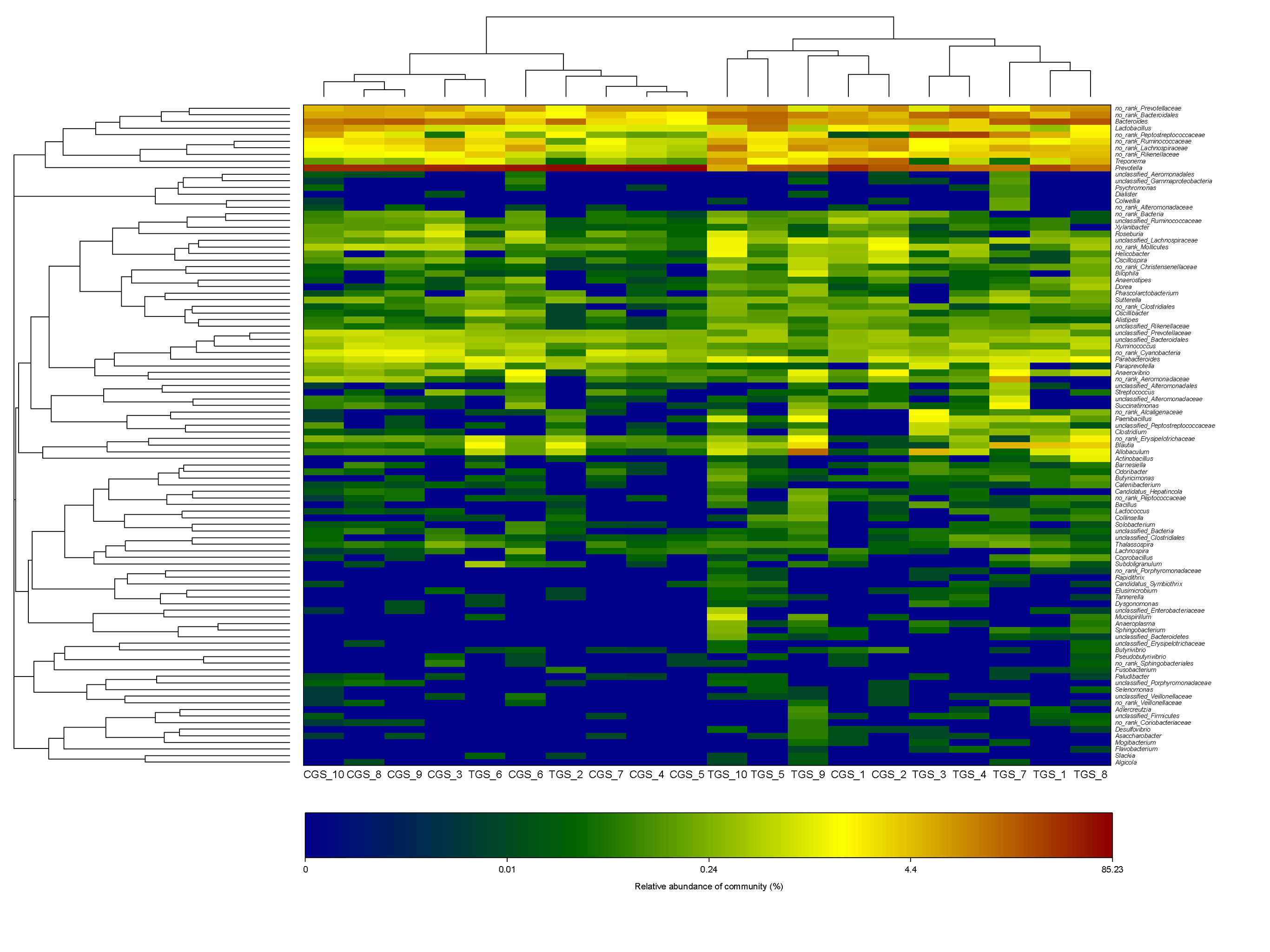

Supplement: Figure S5 — Heatmap analysis of 100 most abundant genera in control group and tumor group. The y axis is a cluster dendrogram, each row is a different phylotype. Clustering is indicative of abundance, not phylogenetic similarity. The abundance plot shows the proportion of 16S rRNA gene pyrosequences in each sample. (TIF) [file pone.0090849.s005.tif]
