# Supplementary material for: Analysis of the Intestinal Lumen Microbiota in an Animal Model of Colorectal Cancer
Source: PLoS One. 2014 Mar 6;9(3):e90849. doi: 10.1371/journal.pone.0090849 (PMC3946251; doi:10.1371/journal.pone.0090849)
Supplement: Table S1 — Mothur diversity indices of bacterial communities in samples from two groups. (DOCX) [file pone.0090849.s006.docx]

**Table S1**. Mothur diversity indices of bacterial communities in samples from two groups

| Sample ID | Reads | 0.03 | | | | | |
| --- | --- | --- | --- | --- | --- | --- | --- |
|  |  | OTU | Ace  (lci, hci) | Chao1  (lci, hci) | Good’s  Coverage | Shannon  (lci, hci) | Simpson  (lci, hci) |
| CGS_1 | 5870 | 1757 | 7467  (7041, 7927) | 4491  (4057, 5007) | 0.801533 | 6.12  (6.07, 6.17) | 0.0109  (0.01, 0.0117) |
| CGS_2 | 7045 | 2103 | 9488  (8984, 10028) | 5432  (4946, 6000) | 0.800000 | 6.34  (6.29, 6.38) | 0.0081  (0.0075, 0.0087) |
| CGS_3 | 5661 | 1728 | 7823  (7390, 8290) | 4448  (4014, 4965) | 0.795972 | 5.86  (5.8, 5.92) | 0.0283  (0.0257, 0.0309) |
| CGS_4 | 8651 | 2125 | 8711  (8252, 9204) | 5039  (4620, 5528) | 0.838687 | 6.07  (6.02, 6.11) | 0.0112  (0.0104, 0.0119) |
| CGS_5 | 8376 | 2027 | 8032  (7603, 8495) | 4777  (4371, 5255) | 0.844795 | 6.01  (5.96, 6.06) | 0.0117  (0.0109, 0.0124) |
| CGS_6 | 7800 | 2417 | 12143  (11563, 12761) | 6231  (5715, 6827) | 0.788974 | 6.46  (6.42, 6.51) | 0.0071  (0.0066, 0.0076) |
| CGS_7 | 7384 | 2376 | 11035  (10478, 11629) | 6554  (5973, 7228) | 0.779523 | 6.5  (6.46, 6.55) | 0.0064  (0.0059, 0.0068) |
| CGS_8 | 6458 | 2045 | 10744  (10175, 11353) | 5608  (5084, 6221) | 0.779653 | 6.17  (6.11, 6.22) | 0.0155  (0.014, 0.017) |
| CGS_9 | 6202 | 1934 | 10232  (9659, 10847) | 5274  (4773, 5864) | 0.782167 | 6.05  (6, 6.11) | 0.0207  (0.0187, 0.0228) |
| CGS_10 | 13132 | 2955 | 12439  (11891, 13020) | 7257  (6720, 7870) | 0.853488 | 6.12  (6.08, 6.16) | 0.0142  (0.0134, 0.015) |
| TGS_1 | 7313 | 1924 | 9198  (8697, 9736) | 5278  (4764, 5886) | 0.820730 | 5.63  (5.57, 5.69) | 0.0411  (0.0378, 0.0444) |
| TGS_2 | 8607 | 2021 | 7547  (7156, 7967) | 4723  (4320, 5196) | 0.851516 | 5.74  (5.69, 5.79) | 0.0216  (0.0203, 0.0229) |
| TGS_3 | 7781 | 2029 | 8911  (8425, 9434) | 5328  (4838, 5904) | 0.824316 | 5.73  (5.67, 5.78) | 0.0287  (0.0266, 0.0308) |
| TGS_4 | 6750 | 1973 | 9951  (9386, 10559) | 5771  (5193, 6452) | 0.794963 | 5.79  (5.72, 5.85) | 0.0456  (0.0417, 0.0496) |
| TGS_5 | 6663 | 1894 | 11474  (10848, 12143) | 5743  (5141, 6457) | 0.799790 | 6.12  (6.07, 6.17) | 0.0106  (0.0097, 0.0114) |
| TGS_6 | 6745 | 2081 | 10300  (9749, 10889) | 5751  (5210, 6384) | 0.787398 | 6.19  (6.13, 6.24) | 0.0162  (0.0146, 0.0177) |
| TGS_7 | 8432 | 1849 | 7852  (7423, 8314) | 4698  (4252, 5227) | 0.856973 | 5.57  (5.52, 5.62) | 0.0288  (0.0268, 0.0308) |
| TGS_8 | 9578 | 1997 | 9417  (8926, 9944) | 4972  (4526, 5497) | 0.863750 | 5.75  (5.71, 5.8) | 0.0242  (0.0224, 0.0261) |
| TGS_9 | 8686 | 2276 | 9674  (9183, 10201) | 5818  (5316, 6403) | 0.827423 | 6.16  (6.11, 6.2) | 0.0134  (0.0124, 0.0144) |
| TGS_10 | 7426 | 2412 | 12421  (11813, 13068) | 6809  (6204, 7511) | 0.774037 | 6.5  (6.46, 6.55) | 0.0068  (0.0063, 0.0073) |

| Sample ID | Reads | 0.05 | | | | | |
| --- | --- | --- | --- | --- | --- | --- | --- |
|  |  | OTU | Ace  (lci, hci) | Chao1  (lci, hci) | Good’s  Coverage | Shannon  (lci, hci) | Simpson  (lci, hci) |
| CGS_1 | 5870 | 1374 | 4783  (4484, 5111) | 3100  (2794, 3473) | 0.854514 | 5.6  (5.54, 5.65) | 0.0172  (0.016, 0.0183) |
| CGS_2 | 7045 | 1674 | 6551  (6172, 6963) | 4244  (3820, 4753) | 0.847268 | 5.83  (5.78, 5.88) | 0.0149  (0.0138, 0.016) |
| CGS_3 | 5661 | 1333 | 5013  (4702, 5352) | 3122  (2799, 3517) | 0.851970 | 5.32  (5.26, 5.39) | 0.0433  (0.0397, 0.0469) |
| CGS_4 | 8651 | 1511 | 4965  (4671, 5286) | 3129  (2855, 3457) | 0.894054 | 5.34  (5.29, 5.39) | 0.0248  (0.0231, 0.0265) |
| CGS_5 | 8376 | 1418 | 4518  (4240, 4822) | 3043  (2756, 3391) | 0.898997 | 5.28  (5.23, 5.33) | 0.0247  (0.0232, 0.0263) |
| CGS_6 | 7800 | 1826 | 7126  (6738, 7544) | 4244  (3868, 4689) | 0.851282 | 5.9  (5.85, 5.94) | 0.0135  (0.0125, 0.0146) |
| CGS_7 | 7384 | 1723 | 6089  (5744, 6464) | 3849  (3511, 4252) | 0.854280 | 5.84  (5.79, 5.88) | 0.0141  (0.0131, 0.0151) |
| CGS_8 | 6458 | 1586 | 6852  (6442, 7297) | 3849  (3478, 4294) | 0.838185 | 5.61  (5.55, 5.66) | 0.025  (0.0229, 0.027) |
| CGS_9 | 6202 | 1489 | 6338  (5934, 6778) | 3652  (3287, 4093) | 0.841180 | 5.5  (5.44, 5.56) | 0.0315  (0.0289, 0.0342) |
| CGS_10 | 13132 | 2194 | 8042  (7649, 8463) | 4739  (4376, 5163) | 0.897883 | 5.51  (5.47, 5.56) | 0.022  (0.0209, 0.023) |
| TGS_1 | 7313 | 1480 | 5635  (5290, 6010) | 3449  (3113, 3855) | 0.870915 | 5.2  (5.14, 5.25) | 0.046  (0.0426, 0.0494) |
| TGS_2 | 8607 | 1448 | 4712  (4433, 5016) | 3150  (2849, 3515) | 0.900546 | 5.09  (5.04, 5.14) | 0.0409  (0.0385, 0.0433) |
| TGS_3 | 7781 | 1614 | 6031  (5677, 6416) | 3752  (3400, 4172) | 0.868140 | 5.3  (5.25, 5.36) | 0.0349  (0.0326, 0.0372) |
| TGS_4 | 6750 | 1605 | 7216  (6789, 7679) | 4081  (3671,4571) | 0.842519 | 5.46  (5.4, 5.52) | 0.049  (0.0449, 0.053) |
| TGS_5 | 6663 | 1478 | 7714  (7244, 8222) | 4020  (3577, 4556) | 0.851268 | 5.65  (5.6, 5.7) | 0.0154  (0.0143, 0.0166) |
| TGS_6 | 6745 | 1566 | 5986  (5618, 6389) | 3717  (3359, 4146) | 0.850556 | 5.66  (5.61, 5.71) | 0.0204  (0.0188, 0.0221) |
| TGS_7 | 8432 | 1433 | 4869  (4573, 5193) | 3246  (2925, 3634) | 0.896466 | 5.11  (5.06, 5.16) | 0.0358  (0.0337, 0.0379) |
| TGS_8 | 9578 | 1503 | 5804  (5445, 6195) | 3534  (3183, 3958) | 0.902380 | 5.29  (5.25, 5.34) | 0.0285  (0.0265, 0.0304) |
| TGS_9 | 8686 | 1820 | 6805  (6425, 7217) | 4463  (4042, 4963) | 0.867603 | 5.7  (5.65, 5.74) | 0.0206  (0.0192, 0.022) |
| TGS_10 | 7426 | 1996 | 8782  (8308, 9291 | 5205  (4722, 5773) | 0.821034 | 6.16  (6.11, 6.2) | 0.0098  (0.0091, 0.0106) |

| Sample ID | Reads | 0.1 | | | | | |
| --- | --- | --- | --- | --- | --- | --- | --- |
|  |  | OTU | Ace  (lci, hci) | Chao1  (lci, hci) | Good’s  Coverage | Shannon  (lci, hci) | Simpson  (lci, hci) |
| CGS_1 | 5870 | 911 | 2500  (2318, 2705) | 1694  (1525, 1909) | 0.914651 | 4.89  (4.83, 4.94) | 0.0337  (0.0316, 0.0358) |
| CGS_2 | 7045 | 1102 | 3069  (2865, 3297) | 2158  (1945, 2425) | 0.914123 | 5.19  (5.14, 5.24) | 0.0287  (0.0266, 0.0309) |
| CGS_3 | 5661 | 880 | 2620  (2429, 2834) | 1789  (1587, 2049) | 0.912383 | 4.66  (4.6, 4.73) | 0.0589  (0.0546, 0.0632) |
| CGS_4 | 8651 | 868 | 2148  (1990, 2329) | 1496  (1357, 1674) | 0.946618 | 4.18  (4.13, 4.23) | 0.0717  (0.0679, 0.0756) |
| CGS_5 | 8376 | 797 | 1850  (1713, 2007) | 1386  (1248, 1566) | 0.951648 | 4.09  (4.04, 4.14) | 0.077  (0.0729, 0.0811) |
| CGS_6 | 7800 | 1122 | 3174  (2965, 3407) | 2161  (1956, 2417) | 0.920000 | 4.91  (4.86, 4.96) | 0.0396  (0.0369, 0.0423) |
| CGS_7 | 7384 | 987 | 2656  (2472, 2863) | 1880  (1692, 2118) | 0.926869 | 4.76  (4.71, 4.81) | 0.0365  (0.0344, 0.0386) |
| CGS_8 | 6458 | 1011 | 2989  (2778, 3224) | 1937  (1748, 2175) | 0.911273 | 4.95  (4.9, 5) | 0.0342  (0.0318, 0.0367) |
| CGS_9 | 6202 | 965 | 2863  (2650, 3104) | 1889  (1697, 2131) | 0.910351 | 4.87  (4.81, 4.93) | 0.042  (0.0389, 0.0452) |
| CGS_10 | 13132 | 1340 | 3549  (3334, 3788) | 2516  (2299, 2782) | 0.944944 | 4.85  (4.81, 4.89) | 0.0294  (0.0282, 0.0306) |
| TGS_1 | 7313 | 993 | 3017  (2801, 3258) | 1964  (1764, 2217) | 0.922057 | 4.51  (4.45, 4.57) | 0.0684  (0.064, 0.0727) |
| TGS_2 | 8607 | 841 | 2357  (2183, 2553) | 1579  (1411, 1798) | 0.948066 | 4.08  (4.02, 4.13) | 0.1057  (0.1002, 0.1111) |
| TGS_3 | 7781 | 1070 | 2860  (2669, 3073) | 2071  (1867, 2329) | 0.924945 | 4.75  (4.7, 4.81) | 0.0467  (0.044, 0.0494) |
| TGS_4 | 6750 | 1086 | 3577  (3336, 3844) | 2394  (2127, 2730) | 0.906815 | 4.93  (4.87, 4.98) | 0.0642  (0.0593, 0.069) |
| TGS_5 | 6663 | 969 | 3571  (3309, 3862) | 2276  (1995, 2635) | 0.913102 | 4.99  (4.94, 5.04) | 0.028  (0.0261, 0.0299) |
| TGS_6 | 6745 | 984 | 3040  (2821, 3286) | 2054  (1830, 2388) | 0.915641 | 4.86  (4.81, 4.91) | 0.0332  (0.0312, 0.0353) |
| TGS_7 | 8432 | 959 | 2571  (2394, 2770) | 1772  (1597, 1995) | 0.939635 | 4.57  (4.53, 4.62) | 0.0505  (0.0478, 0.0531) |
| TGS_8 | 9578 | 993 | 2958  (2744, 3198) | 1972  (1767, 2233) | 0.942055 | 4.7  (4.66, 4.74) | 0.0406  (0.0382, 0.043) |
| TGS_9 | 8686 | 1247 | 3568  (3340, 3820) | 2562  (2310, 2874) | 0.919641 | 5.12  (5.07, 5.16) | 0.0322  (0.0302, 0.0341) |
| TGS_10 | 7426 | 1378 | 4224  (3967, 4507) | 2933  (2650, 3279) | 0.892944 | 5.65  (5.61, 5.7) | 0.0138  (0.0129, 0.148) |

| Sample ID | Reads | unique | | | | | |
| --- | --- | --- | --- | --- | --- | --- | --- |
|  |  | OTU | Ace  (lci, hci) | Chao1  (lci, hci) | Good’s  Coverage | Shannon  (lci, hci) | Simpson  (lci, hci) |
| CGS_1 | 5870 | 3188 | 38364  (36259, 40604) | 16877  (15054, 18981) | 0.541908 | 7.14  (7.09, 7.19) | 0.0049  (0.0043, 0.0054) |
| CGS_2 | 7045 | 3674 | 51993  (49665, 54439) | 19582  (17588, 21862) | 0.561391 | 7.28  (7.23, 7.32) | 0.0034  (0.0031, 0.0037) |
| CGS_3 | 5661 | 2911 | 39531  (37498, 41685) | 17354  (15268, 19793) | 0.561738 | 6.56  (6.49, 6.63) | 0.0242  (0.0218, 0.0266) |
| CGS_4 | 8651 | 4284 | 69752  (66535, 73135) | 25973  (23362, 28942) | 0.568158 | 7.13  (7.08, 7.18) | 0.0057  (0.0052, 0.0061) |
| CGS_5 | 8376 | 4102 | 57937  (55154, 60871) | 22820  (20584, 25360) | 0.580946 | 7.07  (7.02, 7.11) | 0.0062  (0.0057, 0.0067) |
| CGS_6 | 7800 | 4214 | 63554  (60464, 66814) | 27555  (24642, 30883) | 0.532308 | 7.33  (7.29, 7.38) | 0.0039  (0.0036, 0.0042) |
| CGS_7 | 7384 | 4245 | 92900  (88780, 97221) | 32023  (28425, 36157) | 0.491197 | 7.4  (7.35, 7.44) | 0.0036  (0.0033, 0.0039) |
| CGS_8 | 6458 | 3475 | 62398  (59169, 65814) | 24513  (21580, 27922) | 0.528801 | 6.99  (6.94, 7.04) | 0.0097  (0.0086, 0.0107) |
| CGS_9 | 6202 | 3265 | 53033  (50329, 55891) | 19711  (17468, 22309) | 0.545953 | 6.89  (6.83, 6.95) | 0.0124  (0.011, 0.0138) |
| CGS_10 | 13132 | 5852 | 83230  (80001, 86600) | 31051  (28539, 33842) | 0.621154 | 7.08  (7.03, 7.12) | 0.0096  (0.009, 0.0102) |
| TGS_1 | 7313 | 3353 | 57010  (54395, 59758) | 20796  (18414, 23556) | 0.605907 | 6.37  (6.31, 6.44) | 0.0344  (0.0315, 0.0374) |
| TGS_2 | 8607 | 4044 | 62420  (59422, 65580) | 25469  (22778, 28548) | 0.594632 | 6.74  (6.68, 6.79) | 0.0139  (0.0128, 0.0149) |
| TGS_3 | 7781 | 3725 | 49615  (47273, 52082) | 20503  (18405, 22901) | 0.592854 | 6.68  (6.62, 6.74) | 0.0196  (0.0179, 0.0213) |
| TGS_4 | 6750 | 3269 | 47021  (44637, 49543) | 19558  (17323, 22147) | 0.587407 | 6.53  (6.46, 6.59) | 0.0378  (0.0342, 0.0413) |
| TGS_5 | 6663 | 3395 | 55835  (53338, 58458) | 20923  (18530, 23695) | 0.566412 | 7.03  (6.99, 7.08) | 0.0063  (0.0057, 0.0069) |
| TGS_6 | 6745 | 3642 | 66338  (63240, 69597) | 25797  (22756, 29323) | 0.529726 | 7  (6.95, 7.06) | 0.0125  (0.0111, 0.0139) |
| TGS_7 | 8432 | 3542 | 43561  (41467, 45771) | 17623  (15868, 19629) | 0.648956 | 6.42  (6.37, 6.48) | 0.0233  (0.0215, 0.0252) |
| TGS_8 | 9578 | 4002 | 60919  (58382, 63574) | 25615  (22824, 28819) | 0.646586 | 6.68  (6.63, 6.73) | 0.0193  (0.0176, 0.021) |
| TGS_9 | 8686 | 4318 | 50803  (48601, 53115) | 22183  (20119, 24517) | 0.586461 | 7.2  (7.15, 7.24) | 0.0078  (0.0071, 0.0086) |
| TGS_10 | 7426 | 4097 | 54404  (51881, 57060) | 23464  (21112, 26141) | 0.530299 | 7.39  (7.35, 7.43) | 0.0037  (0.0033, 0.004) |

Sample ID: CGS, control group stool; TGS, tumor group stool. Reads: total trimed sequences that divided into the overall OTUs. The number of OTUs, richness estimator(Chao1, Ace), diversity estimator(Shannon, Simpson), and coverage index(Good’s coverage) were calculated at different levels (0.03, 0.05, 0.1 and unique, respectively). OTUs: operational taxonomic units; lci: lower confidence interval, hci: higher confidence interval.
