# Supplementary material for: Analysis of the Intestinal Lumen Microbiota in an Animal Model of Colorectal Cancer
Source: PLoS One. 2014 Mar 6;9(3):e90849. doi: 10.1371/journal.pone.0090849 (PMC3946251; doi:10.1371/journal.pone.0090849)
Supplement: Table S2 — Composition of dominating phyla. (DOCX) [file pone.0090849.s007.docx]

**Table S2**. Composition of dominating phyla

| Phylum | Class | Order | Family | Genus | Total reads | Reads of TGS group |
| --- | --- | --- | --- | --- | --- | --- |
| Actinobacteria | Actinobacteria | no-rank-Actinobacteria | Micrococcaceae | Arthrobacter | 1 | 1 |
|  |  |  | Promicromonosporaceae | Isoptericola | 1 | 0 |
|  |  |  | Mycobacteriaceae | Mycobacterium | 1 | 0 |
|  |  |  | Micrococcaceae | Yaniella | 1 | 1 |
|  | no-rank-Actinobacteria | Coriobacteriales | Coriobacteriaceae | Adlercreutzia | 13 | 13 |
|  |  |  |  | Asaccharobacter | 13 | 9 |
|  |  |  |  | Atopobium | 3 | 2 |
|  |  |  |  | Collinsella | 56 | 53 |
|  |  |  |  | Slackia | 6 | 6 |
|  |  |  |  | no-rank-Coriobacteriaceae | 14 | 11 |
| Bacteroidetes | Bacteroidia | Bacteroidales | Bacteroidaceae | Bacteroides | 18691 | 11636 |
|  |  |  | Marinilabiaceae | Alkaliflexus | 2 | 2 |
|  |  |  |  | no-rank-Marinilabiaceae | 2 | 1 |
|  |  |  | no-rank-Bacteroidales | no-rank-Bacteroidales | 12607 | 9044 |
|  |  |  | Porphyromonadaceae | Barnesiella | 49 | 33 |
|  |  |  |  | Butyricimonas | 68 | 56 |
|  |  |  |  | Candidatus-Azobacteroides | 3 | 2 |
|  |  |  |  | Candidates-Symbiothrix | 18 | 14 |
|  |  |  |  | Dysgonomonas | 14 | 13 |
|  |  |  |  | Odoribacter | 65 | 43 |
|  |  |  |  | Paludibacter | 15 | 8 |
|  |  |  |  | Parabacteroides | 1317 | 792 |
|  |  |  |  | Porphyromonas | 1 | 1 |
|  |  |  |  | Tannerella | 16 | 16 |
|  |  |  |  | no-rank-Porphyromonadaceae | 7 | 7 |
|  |  |  |  | unclassified-Porphyromonadaceae | 16 | 6 |
|  |  |  | Prevotellaceae | Paraprevotella | 377 | 252 |
|  |  |  |  | Prevotella | 62660 | 20424 |
|  |  |  |  | Xylanibacter | 155 | 43 |
|  |  |  |  | no-rank-Prevotellaceae | 9098 | 3987 |
|  |  |  |  | unclassified- Prevotellaceae | 544 | 184 |
|  |  |  | Rikenellaceae | Alistipes | 143 | 73 |
|  |  |  |  | no-rank-Rikenellaceae | 3676 | 2672 |
|  |  |  |  | unclassified-Rikenellaceae | 203 | 151 |
|  |  |  | unclassified-Bacteroidales | unclassified-Bacteroidales | 638 | 312 |
|  | Cytophagia | Cytophagales | Cyclobacteriaceae | Belliella | 1 | 1 |
|  |  |  | Cytophagaceae | Meniscus | 1 | 1 |
|  |  |  | Flammeovirgaceae | Persicobacter | 3 | 2 |
|  |  |  |  | Rapidithrix | 8 | 8 |
|  | Flavobacteria | Flavobacteriales | Flavobacteriaceae | Capnocytophaga | 1 | 1 |
|  |  |  |  | Costertonia | 1 | 0 |
|  |  |  |  | Flavobacterium | 6 | 6 |
|  |  |  |  | Mesonia | 2 | 2 |
|  | no-rank-Bacteroidetes | no-rank-Bacteroidetes | no-rank-Bacteroidetes | no-rank-Bacteroidetes | 1 | 1 |
|  | Sphingobacteriia | Sphingobacteriales | no-rank-Sphingobacteriales | no-rank-Sphingobacteriales | 11 | 4 |
|  |  |  | Saprospiraceae | Aureispira | 1 | 0 |
|  |  |  | Sphingobacteriaceae | Olivibacter | 1 | 0 |
|  |  |  |  | Sphingobacterium | 46 | 44 |
|  |  |  |  | unclassified-Sphingobacteriaceae | 2 | 2 |
|  | unclassified-Bacteroidetes | unclassified-Bacteroidetes | unclassified-Bacteroidetes | unclassified-Bacteroidetes | 25 | 23 |
| Cyanobacteria | no-rank-Cyanobacteria | no-rank-Cyanobacteria | no-rank-Cyanobacteria | no-rank-Cyanobacteria | 864 | 235 |
|  | unclassified-Cyanobacteria | unclassified-Cyanobacteria | unclassified-Cyanobacteria | unclassified-Cyanobacteria | 1 | 1 |
| Deferribacteres | Deferribacteres | Deferribacterales | Deferribacteraceae | Deferribacter | 1 | 0 |
|  |  |  |  | Mucispirillum | 101 | 99 |
| Firmicutes | Bacilli | Bacillales | Bacillaceae | Bacillus | 45 | 43 |
|  |  |  |  | Cerasibacillus | 1 | 0 |
|  |  |  |  | Paucisalibacillus | 2 | 1 |
|  |  |  | Paenibacillaceae | Cohnella | 1 | 1 |
|  |  |  |  | paenibacillus | 496 | 492 |
|  |  |  | Staphylococcaceae | Staphylococcus | 5 | 3 |
|  |  |  | Thermoactinomycetaceae | no-rank-Thermoactinomycetaceae | 1 | 1 |
|  |  |  | unclassified-Bacillales | unclassified-Bacillales | 4 | 3 |
|  |  | Lactobacillales | Carnobacteriaceae | Carnobacterium | 2 | 2 |
|  |  |  |  | Granulicatella | 1 | 0 |
|  |  |  | Enterococcaceae | Vagococcus | 1 | 1 |
|  |  |  | Lactobacillaceae | Lactobacillus | 4647 | 1810 |
|  |  |  | Streptococcaceae | Lactococcus | 44 | 36 |
|  |  |  |  | Streptococcus | 114 | 61 |
|  |  | unclassified-Bacilli | unclassified-Bacilli | unclassified-Bacilli | 2 | 1 |
|  | Clostridia | no-rank-Clostridia | Nerobacterales-Thermoanaerobacteraceae | Ammonifex | 3 | 3 |
|  |  | Clostridiales | Christensenellaceae | Christensenella | 2 | 2 |
|  |  |  |  | no-rank-Christensenellaceae | 175 | 140 |
|  |  |  |  | unclassified-Christensenellaceae | 6 | 2 |
|  |  |  | Clostridiaceae | Anaerobacter | 1 | 1 |
|  |  |  |  | Anaerovirgula | 1 | 1 |
|  |  |  |  | Clostridium | 231 | 221 |
|  |  |  | Eubacteriaceae | Eubacterium | 72 | 117 |
|  |  |  | Lachnospiraceae | Anaerostipes | 139 | 85 |
|  |  |  |  | Blautia | 1760 | 1702 |
|  |  |  |  | Butyrivibrio | 25 | 11 |
|  |  |  |  | Coprococcus | 1 | 1 |
|  |  |  |  | Dorea | 141 | 120 |
|  |  |  |  | Lachnospira | 60 | 14 |
|  |  |  |  | Oribacterium | 1 | 1 |
|  |  |  |  | Pseudobutyrivibrio | 9 | 4 |
|  |  |  |  | Roseburia | 378 | 132 |
|  |  |  |  | no-rank-Lachnospiraceae | 6614 | 3937 |
|  |  |  |  | unclassified-Lachnospiraceae | 605 | 354 |
|  |  |  | no-rank-Clostridiales | Mogibacterium | 9 | 8 |
|  |  |  |  | Tepidimicrobium | 3 | 3 |
|  |  |  |  | no-rank-Clostridiales | 136 | 91 |
|  |  |  | Peptococcaceae | Desulfitobacterium | 2 | 2 |
|  |  |  |  | Pelotomaculum | 3 | 3 |
|  |  |  |  | no-rank-Peptococcaceae | 68 | 52 |
|  |  |  | Peptostreptococcaceae | no-rank-Peptostreptococcaceae | 8244 | 7030 |
|  |  |  |  | unclassified- Peptostreptococcaceae | 146 | 126 |
|  |  |  | Ruminococcaceae | Anaerotruncus | 2 | 1 |
|  |  |  |  | Fastidiosipila | 3 | 3 |
|  |  |  |  | Hydrogenoanaerobacterium | 3 | 3 |
|  |  |  |  | Oscillibacter | 214 | 122 |
|  |  |  |  | Oscillospira | 327 | 149 |
|  |  |  |  | Papillibacter | 2 | 1 |
|  |  |  |  | Ruminococcus | 544 | 217 |
|  |  |  |  | Subdoligranulum | 66 | 58 |
|  |  |  |  | no-rank-Ruminococcaceae | 4928 | 2377 |
|  |  |  |  | unclassified-Ruminococcaceae | 185 | 68 |
|  |  |  | Syntrophomonadaceae | Pelospora | 1 | 1 |
|  |  |  |  | Syntrophomonas | 1 | 0 |
|  |  |  | unclassified-Clostridiales | unclassified-Clostridiales | 71 | 51 |
|  |  |  | Veillonellaceae | Anaerovibrio | 790 | 387 |
|  |  |  |  | Dialister | 12 | 11 |
|  |  |  |  | Megasphaera | 1 | 1 |
|  |  |  |  | Pelosinus | 1 | 1 |
|  |  |  |  | Phascolarctobacterium | 172 | 140 |
|  |  |  |  | Selenomonas | 8 | 6 |
|  |  |  |  | no-rank-Veillonellaceae | 16 | 10 |
|  |  |  |  | unclassified-Velillonellaceae | 12 | 5 |
|  | Erysipelotrichi | Erysipelotrichales | Erysipelotrichaceae | Allobaculum | 2311 | 2255 |
|  |  |  |  | Catenibacterium | 38 | 27 |
|  |  |  |  | Coprobacillus | 56 | 43 |
|  |  |  |  | Solobacterium | 38 | 22 |
|  |  |  |  | no-rank-Erysipelotrichaceae | 664 | 557 |
|  |  |  |  | unclassified-Erysipelotrichaceae | 8 | 7 |
|  | unclassified-Firmicutes | unclassified-Firmicutes | unclassified-Firmicutes | unclassified-Firmicutes | 24 | 19 |
| Proteobacteria | Alphaproteobacteria | Rhizobiales | Bradyrhizobiaceae | Afipia | 1 | 0 |
|  |  |  | Methylobacteriaceae | Methylobacterium | 1 | 1 |
|  |  |  | Rhizobiaceae | Shinella | 1 | 1 |
|  |  | Rhodospirillales | Rhodospirillaceae | Thalassopira | 125 | 76 |
|  |  | Rickettsiales | no-rank-Rickettsiales | Candidates-Captivus | 2 | 1 |
|  |  |  |  | Candidates-Hepatincola | 42 | 27 |
|  | Betaproteobacteria | Burkholderiales | Alcaligenaceae | Achromobacter | 1 | 0 |
|  |  |  |  | Alcaligenes | 1 | 1 |
|  |  |  |  | Castellaniella | 1 | 1 |
|  |  |  |  | Sutterella | 332 | 199 |
|  |  |  |  | no-rank-Alcaligenaceae | 244 | 241 |
|  |  |  | Oxalobacteraceae | Janthinobacterium | 2 | 2 |
|  | Deltaproteobacteria | Desulfovibrionales | Desulfovibrionaceae | Bilophila | 188 | 139 |
|  |  |  |  | Desulfovibrio | 13 | 11 |
|  | Epsilonproteobacteria | Campylobacterales | Helicobacteraceae | Helicobacter | 321 | 203 |
|  |  | Nautiliales | Nautiliaceae | Lebetimonas | 1 | 0 |
|  | Gammaproteobacteria | Aeromonadales | Succinivibrionaceae | Succinatimonas | 265 | 202 |
|  |  |  | Aeromonadaceae | no-rank-Aeromonadaceae | 1048 | 768 |
|  |  |  | unclassified-Aeromonadales | unclassified-Aeromonadales | 21 | 11 |
|  |  | Alteromonadales | Alteromonadaceae | Alteromonas | 5 | 2 |
|  |  |  |  | Marinobacter | 2 | 1 |
|  |  |  |  | Microbulbifer | 16 | 1 |
|  |  |  |  | no-rank-Alteromonadaceae | 22 | 0 |
|  |  |  |  | unclassified-Alteromonadaceae | 137 | 0 |
|  |  |  | Colwelliaceae | Colwellia | 18 | 10 |
|  |  |  |  | Thalassomonas | 3 | 1 |
|  |  |  |  | no-rank-Colwelliaceae | 1 | 0 |
|  |  |  | Pseudoalteromonadaceae | Algicola | 15 | 6 |
|  |  |  | Psychromonadaceae | Psychromonas | 19 | 1 |
|  |  |  | Shewanellaceae | Shewanella | 104 | 2 |
|  |  |  | unclassified-Alteromonadales | unclassified-Alteromonadales | 61 | 47 |
|  |  | Enterobacteriales | Enterobacteriaceae | Dickeya | 1 | 1 |
|  |  |  |  | unclassified-Enterobacteriaceae | 42 | 40 |
|  |  | Pasteurellales | Pasteurellaceae | Actinobacillus | 141 | 141 |
|  |  |  |  | Haemophilus | 1 | 1 |
|  |  |  |  | Pasteurella | 4 | 4 |
|  |  | Pseudomonadales | Moraxellaceae | Acinetobacter | 1 | 1 |
|  |  |  |  | Psychrobacter | 2 | 2 |
|  |  |  | Pseudomonadaceae | Pseudomonas | 4 | 3 |
|  |  | unclassified-Gammaproteobacteria | unclassified-Gammaproteobacteria | unclassified-Gammaproteobacteria | 24 | 16 |
|  |  | Xanthomonadales | Xanthomonadaceae | Thermomonas | 1 | 1 |
| Spirochaetes | Spirochaetes | Spirochaetales | Spirochaetaceae | Spirochaeta | 4 | 4 |
|  |  |  |  | Treponema | 4221 | 1894 |
|  |  |  |  | no-rank-Spirochaetaceae | 1 | 1 |
| Tenericutes | Mollicutes | Acholeplasmatales | Acholeplasmataceae | Acholeplasma | 4 | 4 |
|  |  | Anaeroplasmatales | Anaeroplasmataceae | Anaeroplasma | 40 | 40 |
|  |  | Mycoplasmatales | Mycoplasmataceae | Mycoplasma | 4 | 4 |
|  |  | no-rank-Mollicutes | no-rank-Mollicutes | no-rank-Mollicutes | 647 | 290 |
| unclassified-Bacteria | unclassified-Bacteria | unclassified-Bacteria | unclassified-Bacteria | unclassified-Bacteria | 58 | 32 |
| Fusobacteria | Fusobacteria | Fusobacteriales | Fusobacteriaceae | Fusobacterium | 10 | 10 |
| no-rank-Bacteria | no-rank-Bacteria | no-rank-Bacteria | no-rank-Bacteria | no-rank-Bacteria | 166 | 54 |
| Elusimicrobia | Elusimicrobia | Elusimicrobiales | Elusimicrobiaceae | Elusimicrobium | 12 | 5 |
