# Supplementary material for: Analysis of the Intestinal Lumen Microbiota in an Animal Model of Colorectal Cancer
Source: PLoS One. 2014 Mar 6;9(3):e90849. doi: 10.1371/journal.pone.0090849 (PMC3946251; doi:10.1371/journal.pone.0090849)
Supplement: Table S3 — Phylotypes significantly different between gut microbiota of healthy rats and CRC rats. (DOCX) [file pone.0090849.s008.docx]

**Table S3**. Phylotypes significantly different between gut microbiota of healthy rats and CRC rats.

| Taxonomic rank | | Relative abundance(%)# | | *p*-value |
| --- | --- | --- | --- | --- |
|  |  | CGS | TGS |  |
| phylum | Actinobacteria | 0.02 | 0.12 | <0.001 |
| phylum | Bacteroidetes | 79.26 | 63.95 | 0 |
| genus | Bacteroides | 9.22 | 14.92 | <0.001 |
| genus | no-rank-Bacteroidales | 4.66 | 11.6 | 0 |
| genus | Prevotella | 55.22 | 26.19 | 0 |
| genus | no-rank-Rikenellaceae | 1.31 | 3.43 | <0.001 |
| genus | no-rank-Prevotellaceae | 6.68 | 5.11 | <0.001 |
| genus | unclassified-Prevotellace | 0.47 | 0.24 | <0.001 |
| genus | Parabacteroides | 0.69 | 1.02 | <0.001 |
| genus | unclassified-Rikenellaceae | 0.07 | 0.19 | <0.001 |
| genus | Sphingobacterium | 0 | 0.06 | <0.001 |
| genus | Paraprevotella | 0.16 | 0.32 | <0.001 |
| genus | Xylanibacter | 0.15 | 0.06 | <0.001 |
| genus | Butyricimonas | 0.02 | 0.07 | <0.001 |
| genus | unclassified-Bacteroidetes | 0 | 0.03 | <0.001 |
| genus | Tannerella | 0 | 0.02 | <0.001 |
| genus | Dysgonomonas | 0 | 0.02 | 0.002 |
| genus | Rapidithrix | 0 | 0.01 | 0.008 |
| genus | no-rank-Porphyromonadaceae | 0 | 0.01 | 0.016 |
| genus | Odoribacter | 0.03 | 0.06 | 0.016 |
| genus | Barnesiella | 0.02 | 0.04 | 0.022 |
| genus | Candidatus-Symbiothrix | 0.01 | 0.02 | 0.031 |
| genus | Flavobacterium | 0 | 0.01 | 0.031 |
| phylum | Firmicutes | 15.14 | 29.55 | 0 |
| genus | Blautia | 0.08 | 2.18 | 0 |
| genus | no-rank-Peptostreptococcaceae | 1.59 | 9.02 | 0 |
| genus | Allobaculum | 0.07 | 2.89 | 0 |
| genus | Paenibacillus | 0.01 | 0.63 | <0.001 |
| genus | no-rank-Erysipelotrichaceae | 0.14 | 0.71 | <0.001 |
| genus | Lactobacillus | 3.71 | 2.32 | <0.001 |
| genus | Clostridium | 0.01 | 0.28 | <0.001 |
| genus | no-rank-Lachnospiraceae | 3.5 | 5.05 | <0.001 |
| genus | Unclassified-Peptostreptococcaceae | 0.03 | 0.16 | <0.001 |
| genus | Dorea | 0.03 | 0.15 | <0.001 |
| genus | Phascolarctobacterium | 0.04 | 0.18 | <0.001 |
| genus | no-rank-Christensenellaceae | 0.05 | 0.18 | <0.001 |
| genus | Bacillus | 0 | 0.06 | <0.001 |
| genus | Subdoligranulum | 0.01 | 0.07 | <0.001 |
| genus | Roseburia | 0.32 | 0.17 | <0.001 |
| genus | Ruminococcus | 0.43 | 0.28 | <0.001 |
| genus | no-rank-Peptococcaceae | 0.02 | 0.07 | <0.001 |
| genus | Lactococcus | 0.01 | 0.05 | <0.001 |
| genus | Lachnospira | 0.06 | 0.02 | <0.001 |
| genus | Coprobacillus | 0.02 | 0.06 | <0.001 |
| genus | Eubacterium | 0.01 | 0.07 | <0.001 |
| genus | unclassified-Lachnospiraceae | 0.33 | 0.45 | <0.001 |
| genus | no-rank-Clostridiales | 0.06 | 0.12 | <0.001 |
| genus | unclassified-Ruminococcaceae | 0.15 | 0.09 | <0.001 |
| genus | unclassified-Clostridiales | 0.03 | 0.07 | <0.001 |
| genus | no-rank-Ruminococcaceae | 3.34 | 3.05 | 0.001 |
| genus | Dialister | 0 | 0.01 | 0.006 |
| genus | unclassified-Firmicutes | 0.01 | 0.02 | 0.007 |
| genus | Catenibacterium | 0.01 | 0.03 | 0.014 |
| genus | Anaerostipes | 0.07 | 0.11 | 0.015 |
| genus | Mogibacterium | 0 | 0.01 | 0.039 |
| phylum | Proteobacteria | 1.06 | 2.95 | <0.001 |
| genus | no-rank-Alcaligenaceae | 0 | 0.31 | <0.001 |
| genus | no-rank-Aeromonadaceae | 0.37 | 0.98 | <0.001 |
| genus | Actinobacillus | 0 | 0.18 | <0.001 |
| genus | Succinatimonas | 0.08 | 0.26 | <0.001 |
| genus | Bilophila | 0.06 | 0.18 | <0.001 |
| genus | unclassified-Enterobacteria | 0 | 0.05 | <0.001 |
| genus | unclassified-Alteromonadaceae | 0.04 | 0.13 | <0.001 |
| genus | Helicobacter | 0.15 | 0.26 | <0.001 |
| genus | unclassified-Alteromonadales | 0.02 | 0.06 | <0.001 |
| genus | Sutterella | 0.17 | 0.26 | <0.001 |
| genus | Colwellia | 0 | 0.02 | 0.001 |
| genus | Desulfovibrio | 0 | 0.01 | 0.023 |
| genus | Thalassospira | 0.06 | 0.1 | 0.027 |
| phylum | Cyanobacteria | 0.82 | 0.30 | <0.001 |
| genus | no-rank-Cyanobacteria | 0.82 | 0.3 | <0.001 |
| phylum | Deferribacteres | 0.004 | 0.13 | <0.001 |
| genus | Deferribacter | 0 | 0 | 0.495 |
| genus | Mucispirillum | 0 | 0.13 | <0.001 |
| genus | Collinsella | 0 | 0.07 | <0.001 |
| genus | Adlercreutzia | 0 | 0.02 | <0.001 |
| genus | Slackia | 0 | 0.01 | 0.031 |
| phylum | Spirochaetes | 3.04 | 2.44 | <0.001 |
| genus | Spirochaeta | 0 | 0.01 | 0.125 |
| genus | Treponema | 3.04 | 2.43 | <0.001 |
| phylum | no-rank-Bacteria | 0.15 | 0.07 | <0.001 |
| genus | no-rank-Bacteria | 0.15 | 0.07 | <0.001 |
| phylum | Fusobacteria | 0 | 0.01 | 0.002 |
| genus | Fusobacterium | 0 | 0.01 | 0.002 |

Statistical analysis was performed by Mann-Whitney test.

# Relative abundance were shown as mean. Data had no statistically significant difference were not shown.
